# Heparin-induced thrombocytopenia associated with low-molecular-weight heparin: clinical feature analysis of cases and pharmacovigilance assessment of the FAERS database

**DOI:** 10.3389/fphar.2023.1247253

**Published:** 2023-09-20

**Authors:** Leping Liu, Hong Zheng, Shanshan Chen, Shengfeng Wang, Minghua Yang

**Affiliations:** ^1^ Department of Pediatrics, The Third Xiangya Hospital, Central South University, Changsha, Hunan, China; ^2^ Department of Pediatrics, The Xiangya Hospital, Central South University, Changsha, Hunan, China; ^3^ Department of Pharmacy, Hunan Cancer Hospital, The Affiliated Cancer Hospital of Xiangya School of Medicine, Central South University, Changsha, Hunan, China; ^4^ Department of Pharmacy, The Third Xiangya Hospital, Central South University, Changsha, Hunan, China; ^5^ Postdoctoral Research Station of Clinical Medicine, The Third Xiangya Hospital, Central South University, Changsha, Hunan, China; ^6^ MOE Key Lab of Rare Pediatric Diseases, The Third Xiangya Hospital, Central South University, Changsha, Hunan, China; ^7^ Hunan Clinical Research Center of Pediatric Cancer, The Third Xiangya Hospital, Central South University, Changsha, Hunan, China

**Keywords:** low-molecular-weight heparin, heparin-induced thrombocytopenia, characteristics, treatments, pharmacovigilance

## Abstract

**Background:** Unfractionated heparin (UFH) and low-molecular-weight heparin (LMWH) are commonly used anticoagulants for the management of arterial and venous thromboses. However, it is crucial to be aware that LMWH can, in rare cases, lead to a dangerous complication known as heparin-induced thrombocytopenia (HIT). The objective of this study was to evaluate the pharmacovigilance and clinical features of HIT associated with LMWH, as well as identify treatment strategies and risk factors to facilitate prompt management.

**Methods:** We extracted adverse event report data from the FDA Adverse Event Reporting System (FAERS) database for pharmacovigilance assessment. Case reports on LMWH-induced thrombocytopenia dated up to 20 March 2023 were collected for retrospective analysis.

**Results:** Significantly elevated reporting rates of HIT were shown in adverse event (AE) data of LMWHs in the FAERS database, while tinzaparin had a higher proportional reporting ratio (PRR) and reporting odds ratio (ROR) than other LMWHs, indicating a greater likelihood of HIT. Case report analysis indicated that a total of 43 patients showed evidence of LMWH-induced thrombocytopenia with a median onset time of 8 days. Almost half of the events were caused by enoxaparin. LMWHs were mainly prescribed for the treatment of embolism and thromboprophylaxis of joint operation. Patients with a history of diabetes or surgery appeared to be more susceptible to HIT. Clinical symptoms were mostly presented as thrombus, skin lesion, and dyspnea. Almost 90% of the patients experienced a platelet reduction of more than 50% and had a Warkentin 4T score of more than 6, indicating a high likelihood of HIT. In all patients, LMWHs that were determined to be the cause were promptly withdrawn. Following the discontinuation of LMWHs, almost all patients were given alternative anticoagulants and eventually achieved recovery.

**Conclusion:** LMWH-induced thrombocytopenia is rare but serious, with increased risk in patients with diabetes or a surgical history. Prompt recognition and management are crucial for the safe use of LMWHs.

## Introduction

Unfractionated heparin (UFH) and low-molecular-weight heparin (LMWH) have been extensively studied and have demonstrated efficacy in the treatment of arterial and venous thromboses. They are commonly prescribed for conditions such as deep vein thrombosis, pulmonary embolism, acute coronary syndrome, and prophylaxis in high-risk surgical procedures (Burness and Perry, 2014; Ortel et al., 2020). Heparin-induced thrombocytopenia (HIT) is a potentially life-threatening complication that can occur after exposure to UFH, less commonly with LMWHs, and rarely with fondaparinux (Linkins et al., 2012). HIT has been estimated to occur in approximately 1%–5% of patients receiving therapeutic doses of heparin. Additionally, it has been observed that up to 1 in 1,500 hospitalized patients may suffer from HIT (Greinacher, 2015; McGowan et al., 2016; Rice, 2017; Dhakal et al., 2018; Hogan and Berger, 2020; Nilius et al., 2023). HIT caused by UFH is approximately 10-fold higher than that caused by LMWHs. HIT is an immune-mediated reaction caused by the formation of antibodies against the complex of platelet factor 4 (PF4) and heparin. These antibodies can activate platelets to a hypercoagulable state, which resulted in thrombocytopenia and increased the risk of venous and arterial thromboses (Rollin et al., 2022). The most common complications associated with heparin are bleeding, allergic reaction, and osteoporosis (Schindewolf et al., 2012; Signorelli et al., 2019), while thrombocytopenia is a rare complication that often goes unnoticed, especially when it is associated with LMWHs. HIT remains a significant cause of morbidity and mortality in hospitalized patients exposed to heparin, particularly those undergoing cardiac and surgical procedures (Linkins, 2015).

In recent years, increasing efforts have been made to improve the diagnosis and management of HIT. Advances in laboratory testing, including enzyme-linked immunosorbent assays (ELISAs) and functional assays, have improved the specificity and sensitivity to HIT antibodies. In addition, novel non-heparin anticoagulants, such as direct oral anticoagulants (DOACs), have been developed as alternative therapies for HIT. However, despite these advances, HIT remains a challenging diagnosis that requires a high index of suspicion and prompt initiation of appropriate therapy to reduce the risk of thromboembolic complications. The importance of early recognition and appropriate management of HIT cannot be overstated, as delays in diagnosis and treatment can result in terrible consequences, including limb loss, organ damage, and even death (Pishko et al., 2019).

To date, there is a paucity of data regarding HIT associated with LMWHs. In our study, we extracted adverse event report data from the FAERS database for pharmacovigilance assessment and subsequently collected case reports on LMWH-induced thrombocytopenia for a real-world retrospective analysis. We summarized clinical features, risk factors, management, and outcomes of patients with HIT after anticoagulation with LMWHs, which will provide valuable information for the prompt recognition and management of HIT.

## Methods

### Pharmacovigilance study

#### Data extraction from the FAERS database

The FAERS database, which is the drug adverse event reporting system of the US FDA, collects adverse event report data for various drugs, providing strong evidence for drug safety and pharmacovigilance. In order to assess the safety of LMWHs and evaluate the risk of their adverse events (AEs) in clinical use, we retrieved and extracted AE data reported between Quarter 1(Q1) in 2004 and Q3 in 2022 for seven types of LMWHs (enoxaparin, nadroparin, dalteparin, tinzaparin, bemiparin, reviparin, and parnaparin) from the data publicly released by the FAERS database (FDA, 2023).

### Data analysis of the FAERS database

After extraction, the data were initially utilized for baseline analysis, which included variables like gender, age, outcomes, and reporter country. The AEs extracted were then classified according to the Medical Dictionary for Regulatory Activities (MedDRA, Version 26.0), which included categories such as vascular and lymphatic disorders, gastrointestinal disorders, infections and infestations, respiratory disorders, and metabolic and nutritional disorders, among others. We selected HIT among AEs related to vascular and lymphatic disorders and analyzed the association of these LMWHs with HIT by calculating the proportional reporting ratio (PRR) and reporting odds ratio (ROR). In this process, the keywords of PT were set as “heparin-induced thrombocytopenia,” “heparin-induced thrombocytopenia test,” and “heparin-induced thrombocytopenia test positive.”

### Disproportionality analysis

In pharmacovigilance assessment, disproportionality emerges when a specific AE is induced by a given drug. We used PRR and ROR to identify the statistical associations between LMWHs and HIT. PRR and ROR are calculated using the following formulas: PRR = [a/(a+b)]/[c/(c + d)]; ROR = (a/c)/(b/d). In the formula, “a” represents the number of reports of a specific AE caused by the given drug; “b” represents the total number of all other AEs related to the given drug; “c” represents the number of reports of a specific AE caused by all other drugs; and “d” represents the total number of all other AEs related to all other drugs (Supplementary Table S1). For PRR, the screening criteria for an AE defined as a significant signal were PRR >2, χ^2^ > 4, and N >2. For ROR, the screening criteria for a significant signal were the lower limit of two-sided 95% confidence interval (CI) > 1 and N >2.

### Descriptive study

#### Search strategy

Using PubMed/MEDLINE, Web of Knowledge, Embase, Ovid, Springer Link, Elsevier, Cochrane Library, China National Knowledge Infrastructure (CNKI), Wanfang Data, and Chinese VIP databases, our searches for relevant literature were performed using the following keywords: “enoxaparin,” “bemiparin,” “edoxaban,” “nadroparin,” “tinzaparin,” “reviparin,” “parnaparin,” “low-molecular-weight heparin,” “heparin-induced thrombocytopenia,” “heparin related thrombocytopenia,” “anticoagulant-related HIT,” “anticoagulant-related HIT,” “anticoagulant associated HIT,” and “HIT.” Case reports and case series of HIT associated with LMWHs were included as preliminary studies. Duplicate literature, reviews, mechanism research, observational studies, animal studies, and articles without full text were excluded. We conducted thorough searches of various electronic databases without any specified start date up to 20 March 2023 and with no language restrictions.

### Data extraction

We used self-designed tables to extract various clinical features of the patients from cases. These features included gender, age, anticoagulant administration, medical history, combined medication, baseline platelets or PLT, clinical manifestations of HIT, coagulation function, liver and kidney function, the time from taking LMWHs to HIT diagnosed, peak PLT at HIT diagnosis, anti-PF4 antibody, Warkentin 4T score, platelet recovery time, treatments, and prognosis. Specifically, the Warkentin 4T score in HIT incorporates four criteria: extent of platelet reduction, timing of platelet reduction, presence of thrombosis, and other causes of platelet reduction. Each criterion is assigned a score ranging from 0 to 2 points, with a higher cumulative score indicating a greater likelihood of HIT.

## Results

### Pharmacovigilance assessment of the FAERS database

#### Baseline information of HIT related to LMWHs

We retrieved information about three out of seven LMWHs available in the FAERS database: enoxaparin, dalteparin, and tinzaparin. As the FAERS database only recorded adverse events of HIT related to these three LMWHs, our analysis focused on these drugs. The AE data of three types of LMWHs in the FAERS database help comprehensively understand the safety profiles of these drugs. The overall number of the reported AEs of enoxaparin, dalteparin, and tinzaparin was 242, 34, and 30, respectively. Most AEs of the three LMWHs occurred in people aged over 60, ranging between 50% and 70%. The proportion of AEs in male and female patients is similar. Among the AEs of enoxaparin and tinzaparin, male patients accounted for more than 50%, and in the AEs of dalteparin, female patients accounted for more than 50%. The LMWH with the highest proportion of deaths among the outcomes of AEs was tinzaparin, accounting for 36.67%, while the lowest was enoxaparin, accounting for 21.90%. Enoxaparin had the highest proportion of reported cases in the United States, accounting for 38.43% (Table 1).

**TABLE 1:** Baseline table of three types of LMWHs.

	Enoxaparin		Dalteparin		Tinzaparin	
	No. of HIT-associated AEs	Percentage	No. of HIT-associated AEs	Percentage	No. of HIT-associated AEs	Percentage
All	242		34		30	
Age						
<18	2	0.83%	0	0.00%	0	0.00%
18–60	66	27.27%	7	20.59%	7	23.33%
>60	143	59.09%	23	67.65%	16	53.33%
Unknown	31	12.81%	4	11.76%	7	23.33%
Gender						
Female	97	40.08%	18	52.94%	13	43.33%
Male	124	51.24%	15	44.12%	16	53.33%
Unknown	21	8.68%	1	2.94%	1	3.33%
Outcome of events						
Death	53	21.90%	8	23.53%	11	36.67%
Hospitalization-initial or prolonged/disability/life-threatening	157	64.88%	20	58.82%	14	46.67%
Others	32	13.22%	6	17.65%	5	16.67%
Reporter country						
US	93	38.43%	7	20.59%	1	3.33%
Other countries	149	61.57%	27	79.41%	29	96.77%

### PRR and ROR for LMWHs

Since HIT related to nadroparin, bemiparin, reviparin, and parnaparin was not reported in the FAERS database, we only obtained PRR and ROR values of HIT for the other three drugs. PRR and ROR are used to measure the likelihood of a specific AE occurring with a drug, with higher values indicating a stronger association between the drug and the given AE. The PRR values of HIT for enoxaparin, dalteparin, and tinzaparin were 98.22, 100.48, and 195.81, and the ROR values for these three LMWHs were 100.51, 103.10, and 206.06, respectively. Tinzaparin had higher PRR and ROR values, indicating a greater likelihood of HIT occurring with this drug compared to the other two LMWHs (Table 2).

**TABLE 2:** PRR and ROR values of heparin-induced thrombocytopenia.

	χ^2^	PRR (95% CI^a^)	ROR (95% CI^a^)
Enoxaparin	21,060.04	98.22 (86.17, 111.94)	100.51 (87.94, 114.88)
Dalteparin	3,208.847	100.48 (71.95, 140.33)	103.10 (73.19, 145.24)
Tinzaparin	5,558.40	195.81 (137.86, 278.11)	206.06 (142.45, 298.06)

PRR, proportional reporting ratio; ROR, reporting odds ratio; CI, confidence interval.

^a^ Two-sided CI for ROR.

### Clinical feature analysis of cases

#### Patients’ information

**FIGURE 1:**
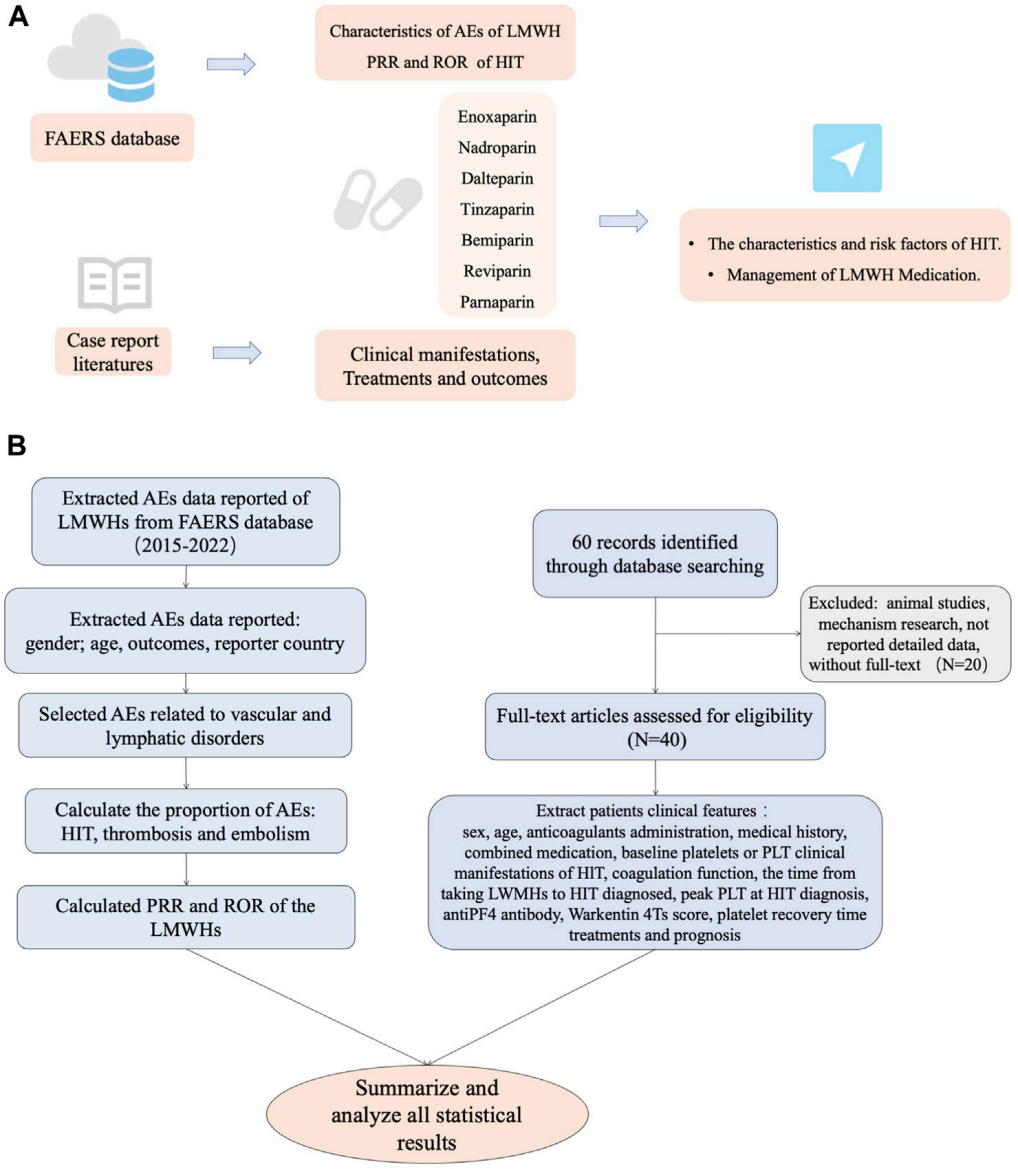
**(A)** Workflow of this research. **(B)** Technical route of this research.

A total of 43 patients from 40 studies were included in this analysis after full-text screening, involving 39 case reports (Plassat et al., 2002; Betrosian et al., 2003; Franke et al., 2003; Ng and Lee, 2003; Zamir et al., 2003; Dager and White, 2004; Patel and Knight, 2005; Doboszyńska et al., 2007; Rota et al., 2008; Famularo et al., 2009; Mumoli and Cei, 2010; Fesler et al., 2011; Illes et al., 2011; Iturbe et al., 2011; Yazbek et al., 2012; Klinkert et al., 2013; Leporini et al., 2013; Nazliel et al., 2014; Sinan et al., 2014; Brouns and Jie, 2015; Giuliani et al., 2015; Hantson et al., 2015; Koufakis et al., 2015; Larsen et al., 2015; Martinez and Burnett, 2015; Sartori et al., 2015; Wiegele et al., 2015; Pérez et al., 2016; Gan, 2017; Rivera et al., 2017; Le et al., 2019; Barcellona et al., 2020; Polák et al., 2020; Singh et al., 2020; Lovatt and Crowther, 2021; Tucker and Padarti, 2021; Byrne et al., 2022; Lázaro-García et al., 2022; Malinauskiene et al., 2022) and one case series (Hartman et al., 2006) (Figure 1). Patients’ information is summarized in Table 3. These patients all had type II HIT. The median age of 43 patients (12 men and 31 women) with HIT in our study was 67 years, with an age range of 11–87 years. The types of LMWH administered were enoxaparin in 21 patients, nadroparin in 14 patients, dalteparin in four patients, tinzaparin in two patients, and bemiparin in two patients. LMWHs were mainly prescribed for embolism (10/43), joint operation (9/43), fracture (5/43), surgery (5/43), and dialysis (4/43). The common medical history in the patients with HIT was diabetes (10/43), surgical history (9/43), obesity (6/43), hypertension (6/43), and tumor history (4/43). As for dosage, all patients were handled in strict accordance with clinical guidelines, and there was no excessive anticoagulation.

**TABLE 3:** Characteristics of the 43 included patients with HIT induced by LMWHs.

Parameter	Value	Percentage (%)
Gender		
Male	12	27.91
Female	31	72.09
Age (years)		
<18	1	2.33
18–60	15	34.88
>60	27	62.79
Low-molecular-weight anticoagulant		
Enoxaparin	21	48.84
Nadroparin	14	32.56
Dalteparin	4	9.30
Tinzaparin	2	4.65
Bemiparin	2	4.65
Primary disease		
Embolism	10	23.26
Joint operation	9	20.93
Fracture	5	11.63
Surgery	5	11.63
Dialysis	4	9.30
Tumor	3	6.98
Heart failure	3	6.98
Traffic accident	2	4.65
Infection	2	4.65
Pregnancy	1	2.33
Medical history		
Diabetes	10	23.26
Surgery	9	20.93
Obesity	6	13.95
Hypertension	6	13.95
Tumor	4	9.30
Chronic renal insufficiency	3	6.98
Myocardial disease	3	6.98
Cholecystectomy	1	2.33
Immune-related diseases	1	2.33
Thrombus history	1	2.33
No risk factors	9	20.93
Combined drugs		
Antihypertensive drugs	4	9.52
Antidiabetic drugs	2	4.76
Antibiotics	2	4.76
Chemotherapeutic drugs	1	2.38
Other types of anticoagulants	1	2.38
Without other drugs	36	85.71

### Clinical manifestations

The clinical characteristics of HIT presented by the 43 included patients are summarized in Table 4. The time between the administration of LMWHs and the onset of HIT varied from 1 day to 30 days, with a median of 8 days. The onset time of HIT and its related outcomes differed based on the type of LMWH administered. The majority of patients (24/43) with HIT presented a typical fashion within 5–10 days. Among these patients, 13 had been administered enoxaparin (a total of 21 patients) while 10 had been given nadroparin (a total of 14 patients). Thrombus (39/43) was the most common clinical symptom, followed by skin lesions (15/43), dyspnea (13/43), hemorrhage (9/43), limb necrosis (5/43), cerebral infarction (5/43), and heart failure (5/43). Some patients may experience severe or even life-threatening symptoms, while others may only have very mild symptoms. We observed three cases (two from enoxaparin and one from nadroparin) of HIT with thrombosis involving multiple organs, including cerebral, pulmonary, abdominal, and lower extremity vessels (Betrosian et al., 2003; Barcellona et al., 2020; Tucker and Padarti, 2021). In contrast, only one case exhibited the symptom of thrombocytopenia (Franke et al., 2003).

**TABLE 4:** Clinical manifestations of HIT induced by LMWHs.

Parameter	Value	Percentage (%)
The time from taking LMWHs to HIT diagnosed (*n* = 43)		
<5 days	5	11.63
Enoxaparin	3	6.98
Nadroparin	1	2.33
Tinzaparin	1	2.33
5–10 days	24	55.81
Enoxaparin	13	30.23
Nadroparin	10	23.26
Bemiparin	1	2.33
>10 days	14	32.56
Enoxaparin	5	11.63
Dalteparin	4	9.30
Nadroparin	3	6.98
Bemiparin	1	2.33
Tinzaparin	1	2.33
Clinical manifestations of HIT (n = 43)		
Thrombus	39	90.70
Skin lesions	15	34.88
Dyspnea	13	30.23
Hemorrhage	9	20.93
Limb necrosis	5	11.63
Cerebral infarction	5	11.63
Heart failure	5	11.63
Acute renal failure	2	4.65
Shock	1	2.33
Fever	1	2.33
Peak PLT at HIT diagnosis		
PLT count (10^^9^/L) (n = 40)		
<10	1	2.50
Nadroparin	1	2.50
10–19	3	7.50
Enoxaparin	1	2.50
Dalteparin	1	2.50
Nadroparin	1	2.50
20–49	13	32.50
Enoxaparin	8	20.00
Nadroparin	2	5.00
Dalteparin	1	2.50
Bemiparin	1	2.50
Tinzaparin	1	2.50
50–100	14	35.00
Enoxaparin	7	17.50
Nadroparin	5	12.50
Dalteparin	1	2.50
Bemiparin	1	2.50
>100	9	22.50
Enoxaparin	5	12.50
Nadroparin	2	5.00
Dalteparin	1	2.50
Tinzaparin	1	2.50
Percentage of PLT decrease (n = 37)		
<30%	1	2.70
Tinzaparin	1	2.70
30%–50%	3	8.11
Enoxaparin	2	5.41
Nadroparin	1	2.70
>50%	33	89.19
Enoxaparin	19	51.35
Nadroparin	8	21.62
Dalteparin	3	8.11
Bemiparin	2	5.41
Tinzaparin	1	2.70
Anti-PF4 antibody(n = 36)		
Positive	36	100.00
Enoxaparin	17	47.22
Nadroparin	12	33.33
Dalteparin	4	11.11
Bemiparin	2	5.56
Tinzaparin	1	2.78
Warkentin 4T score (n = 21)		
0–3	0	0.00
4–5	3	14.29
Dalteparin	2	9.52
Enoxaparin	1	4.76
6–8	18	85.71
Nadroparin	9	42.86
Enoxaparin	6	28.57
Bemiparin	2	9.52
Tinzaparin	1	4.76
D-Dimer elevated (*n* = 11)	5	45.45
Abnormal liver and kidney functions (*n* = 8)	5	62.50

All patients experienced a significant acute decrease in platelet (PLT) count compared to their baseline, with 77.5% of patients having a PLT count of less than 100 × 10^^9^/L. In a subset of cases, the decrease in PLT count was even more severe, with four patients having a PLT count below 20 × 10^^9^/L. Additionally, nearly 90% of patients had a PLT reduction ratio greater than 50%. The lowest PLT count and the proportion of PLT decrease varied among different drugs (Table 4). Among the cases analyzed, enoxaparin accounted for 15 out of 21 cases with PLT counts falling between 20 and 100, while nadroparin accounted for seven out of 14 cases. Regarding the proportion of PLT decrease, enoxaparin accounted for 19 out of 21 cases with a decrease of more than 50%, nadroparin accounted for eight out of 14 cases, and dalteparin accounted for three out of four cases. A total of 36 patients tested positive for anti-PF4 antibodies. Specifically, among these patients, 17 tested positive for enoxaparin (out of a total of 21), 12 tested positive for nadroparin (out of a total of 14), and four tested positive for dalteparin (out of a total of four). The Warkentin 4T scores of 18 patients (85.71%) were more than 6, meaning a high likelihood of HIT. Furthermore, additional laboratory examinations were conducted in some cases. Out of the total cases, 11 patients underwent D-dimer testing and five of them showed elevated D-dimer levels (Betrosian et al., 2003; Dager and White, 2004; Singh et al., 2020; Lovatt and Crowther, 2021; Tucker and Padarti, 2021). Elevated D-dimer levels can be indicative of the ongoing thrombotic activity. Additionally, five out of eight cases showed abnormal liver and kidney functions, which may be associated with HIT.

### Treatments and outcomes

The treatments of HIT were recommended to immediately stop heparin therapy and initiate non-heparin-based anticoagulants. In our included cases, LMWHs, judged as the culprits, were immediately withdrawn in all patients (Table 5). Almost all patients (39/43) were administered other anticoagulants, which included fondaparinux (17/39), lepirudine (11/39), argatroban (7/39), warfarin (5/39), acenocoumarol (5/39), rivaroxaban (4/39), apixaban (2/39), dabigatran (1/39), and clopidogrel (1/39). Two patients with severe systemic symptoms received PLT transfusion. In addition, one patient with SLE was administered glucocorticoid, and one patient with extensive sinus thrombosis was administered a thrombolytic agent. The PLT count recovered within 1 month in all patients, three-quarters (26/34) of whom recovered within 10 days. Finally, almost all patients (41/43) showed recovery; however, one patient died of extensive sinus thrombosis and cerebral hernia (Fesler et al., 2011), and one patient worsened (Betrosian et al., 2003).

**TABLE 5:** Treatments and outcomes of HIT induced by LMWHs.

Parameter	Value	Percentage (%)
Treatment		
Withdraw LMWHs (*n* = 43)	43	100.00
Surgical thrombectomy (*n* = 43)	6	13.95
Emergency treatment (*n* = 43)	1	2.33
Other relevant therapeutic drugs		
Other anticoagulants (*n* = 39)		
Fondaparinux	17	43.59
Lepirudin	11	28.21
Argatroban	7	17.95
Warfarin	5	12.82
Acenocoumarol	5	12.82
Rivaroxaban	4	10.26
Apixaban	2	5.13
Dabigatran	1	2.56
Clopidogrel	1	2.56
Platelet transfusion (*n* = 43)	2	4.65
Thrombolytic agent (*n* = 43)	1	2.33
Glucocorticoid (*n* = 43)	1	2.33
PLT recovery time (*n* = 34)		
<5 days	9	26.47
Nadroparin	4	11.76
Enoxaparin	3	8.82
Dalteparin	2	5.88
5–10 days	17	50.00
Enoxaparin	11	32.35
Nadroparin	4	11.76
Bemiparin	1	2.94
Tinzaparin	1	2.94
>10 days	8	23.53
Nadroparin	5	14.71
Enoxaparin	2	5.88
Bemiparin	1	2.94
Prognosis (*n* = 43)		
Recovery	41	95.35
Worse	1	2.33
Death	1	2.33

## Discussion

Concerns have been raised regarding the risk of HIT in patients exposed to UFH, while there are limited data on HIT associated with LMWHs in recent years. HIT induced by LMWHs is often overlooked. This study investigated the characteristics of the AEs caused by three types of LMWHs in the FAERS database, as well as the reporting rate for HIT events. Additionally, we conducted a literature review on the cases of HIT caused by LMWHs to further understand the features, treatments, and prognoses of these patients. The results from the literature review and the FAERS database analysis showed some similarities to a certain extent. Specifically, in terms of age, the proportion of AE reports involving LMWHs in the database was similar to that reported in the literature, with a higher proportion observed in older individuals (over 60 years of age). Regarding outcomes, the reporting rates of death events associated with the three types of LMWHs in the database ranged from 20% to 37%. Similarly, out of the 43 reported cases of HIT, one resulted in death, one worsened, and 41 patients recovered. These findings suggest that with appropriate clinical intervention, most cases of HIT can be successfully treated and resolved. Furthermore, the analysis of the database revealed that tinzaparin had the highest PRR and ROR values for HIT, indicating a potentially higher likelihood of causing HIT compared to other LMWHs. On the other hand, based on the case literature reports, enoxaparin was found to be associated with the highest number of HIT cases.

HIT is a rare and serious AE caused by heparin, which can be divided into type I and type II. Type I is an early-onset, mild thrombocytopenia that does not lead to thromboembolism, whereas type II is immune-mediated and clinically severe, causing both thrombocytopenia and thromboembolism with approximately 50% of patients developing arterial or venous thrombosis usually occurring 5–10 days after starting heparin therapy (Nazliel et al., 2014; Barcellona et al., 2020; Singh et al., 2020). LMWH has been widely used to prevent thromboembolism because of its safety and ease of administration. HIT caused by UFH is often overlooked in clinical practice due to its low incidence rate (2.6%). In contrast, the incidence rate of HIT caused by LMWH is approximately 0.2%, making it even more likely to be overlooked and not receive timely and effective intervention (Sahu et al., 2020). If patients are not treated in time, serious outcomes may occur, such as amputation, myocardial infarction, pulmonary embolism, cerebral infarction, and even death.

The immunopathological mechanism underlying the development of HIT is still unclear. PLT factor 4 (PF4) is a positively charged protein released from the α-granules of activated platelets and combines with the negatively charged heparin through the electrostatic interaction of the vascular epidermis to form a complex (Joglekar et al., 2015). Immunoglobulin G (IgG) antibodies against the PF4/heparin complex play a major role in the development of HIT II. Approximately 1%–8% of patients receiving LMWH produce these antibodies, only a small portion of which result in thrombosis (Warkentin et al., 2000; Arepally and Hursting, 2008). These antibodies bind to the complex and platelet Fcg receptor IIa, respectively, through their Fab and Fc sequences, activate platelets, generate procoagulant microparticles, and increase the production of thrombin. Immune complexes induce platelet aggregation, resulting in thrombocytopenia. At the same time, they also induce monocytes to express the procoagulant tissue factor, further promoting thrombus formation (Arepally and Mayer, 2001).

In this study, the primary causes of the use of LMWH were mainly the treatment of thrombosis and the thromboprophylaxis of joint operation, fractures, surgical procedures, and dialysis. In addition patients are often accompanied by a history of diabetes, surgery, obesity, hypertension, or tumors. These factors deserve special attention from clinical physicians. Greinacher et al. (2005) found that orthopedic surgery was the most critical factor causing thrombosis in 408 suspected HIT patients. Barcellona et al. (2020) reported a female patient who underwent left knee replacement with urosepsis and developed severe HIT, resulting in cerebral, pulmonary, hepatic, and lower extremity arterial and venous thromboses after receiving LMWH. Studies have shown that obese patients have more fat cells, which are more likely to activate inflammatory pathways, lead to cytokine imbalance, and increase the risk of HIT (Cave et al., 2008). There are also studies that believe that obese patients are prone to skin necrosis due to heparin residues in the subcutaneous fat tissue with poor blood circulation. This may also explain the higher risk of skin necrosis in female patients than in male patients, since female individuals have higher levels of body fat than male individuals (Gan, 2017). Obesity may also contribute to treatment failure due to pharmacokinetics. Larissa et al. reported a case of HIT in a morbidly obese female patient taking enoxaparin. She still experienced extensive thrombosis after 7 days of daily oral 10 mg fondaparinux replacement therapy, which may be due to obesity affecting the absorption of fondaparinux (Martinez and Burnett, 2015). Since HIT is an immune-mediated disease and PF4 is involved in the inflammatory process, Gram-negative bacterial infections can activate the immune system and increase the risk of HIT (Krauel et al., 2012). Skin necrosis is a rare symptom caused by LMWH and usually occurs at the injection site but can also occur elsewhere, primarily in female patients with a history of diabetes and thromboembolic disease (Lubenow et al., 2010). However, it has also been suggested that this skin injury may be caused by delayed type IV hypersensitivity rather than microvascular injury produced by HIT (Schindewolf et al., 2010). The specific mechanism still needs further research. For patients with the aforementioned primary causes and medical history, clinicians should closely monitor their clinical manifestations and laboratory tests after using LMWHs. The focus of laboratory tests is PLT count and HIT antibody.

Although the prognosis of HIT is relatively good, the mortality rate can be as high as 20%–30%, if timely diagnosis and intervention are not employed. It is extremely important to closely monitor the PLT count to prevent the occurrence of HIT (Lassen et al., 2010). It is suggested that for all patients receiving heparin treatment, the baseline PLT count should be monitored before the treatment begins and then every 2–3 days between the fourth and fifteenth day (Linkins et al., 2012). Since IgG antibodies in HIT have a unique transient property and can disappear in 50–80 days, it is recommended that patients exposed to LMWHs within 3 months continue to monitor their PLT counts (Lubenow et al., 2002). Rarely, IgG may persist in circulation for more than 80 days, as reported in an obese patient developing HIT 2 years after the initial exposure to enoxaparin, but this is exceedingly rare (Wiegele et al., 2015). The current guidelines recommend that after the HIT onset, LMWH should be discontinued immediately and replaced with argatroban, bivalirudin, or fondaparinux (Cuker et al., 2018). Studies have shown that rivaroxaban is a safe and effective oral agent for the treatment of HIT and can be used as a replacement for heparin in the prevention of deep vein thrombosis in adults, which is more easily accepted by patients than parenteral administration and requires no laboratory monitoring (Joseph et al., 2014; Le et al., 2019).

This study is the first drug safety study that combines the FAERS database and case reports to study HIT induced by LMWHs. The advantage of this study is that it makes full use of the database and literature resources and comprehensively collects and analyzes a large amount of relevant data, so as to deepen the understanding of the occurrence and characteristics of HIT. This study can provide some guidance for improving clinical prognosis; however, there are certain limitations. First, although the sample size is relatively large, the data are not complete because the FAERS database and literature cases can only provide an analysis of the existing data and cases, ignoring unreported or unprocessed information, so the results may have selection bias. Second, this method cannot directly explain the mechanism underlying the development of HIT caused by LMWHs and requires specific mechanisms to be studied through cell and animal experiments. Third, the lack of multivariable analyses here to implicate the specific role of LMWH in the development of HIT while controlling for other clinical factors is a major limitation to the study. Lastly, prospective or randomized controlled trials are needed to elucidate the relevant characteristics of HIT induced by LMWH administration.

## Conclusion

HIT is a rare but potentially fatal complication of LMWHs. Due to its low incidence and difficult diagnosis, it is often overlooked by clinicians, thus leading to serious outcomes without timely and effective intervention. However, adverse outcomes can be completely avoided by strengthening the understanding of the disease. Our research found that patients who are obese, diabetic, infected, or having a history of surgery, hypertension, or tumor are more likely to develop HIT during LMWH anticoagulation. The pathological mechanism underlying the development of HIT is complex, and the most obvious pathological features are thrombocytopenia and thrombosis. The current guidelines recommend close monitoring of PLT changes and timely HIT antibody testing, if HIT is suspected after LMWH administration. These are critical for treatment and prognosis. In the future, further prospective studies are needed to clarify the risk factors, pathological mechanisms, and related treatments of HIT.

## Data Availability

The original contributions presented in the study are included in the article/Supplementary Material. Further inquiries can be directed to the corresponding authors.
