# Supplementary material for: Heparin-induced thrombocytopenia associated with low-molecular-weight heparin: clinical feature analysis of cases and pharmacovigilance assessment of the FAERS database
Source: Front Pharmacol. 2023 Sep 20;14:1247253. doi: 10.3389/fphar.2023.1247253 (PMC10552922; doi:10.3389/fphar.2023.1247253)
Supplement: Supplementary file 1 [file Table1.docx]

**Supplementary Table 1 Two-by-two contingency table for disproportionality analyses**

|  | Number of reports of target adverse events | Number of reports of other adverse events | Total |
| --- | --- | --- | --- |
| Target drug | a | b | a + b |
| Other drugs | c | d | c + d |
| Total | a + c | b + d | N = a + b + c + d |

The calculation formula for PRR and ROR are as follows:

PRR = [a / (a + b) ] / [c / (c + d)]

ROR = (a / c) / (b / d)

95%*CI* = e^lnROR ± 1.96^ $\sqrt{\frac{1}{a}+\frac{1}{b}+\frac{1}{c}+\frac{1}{d}}$

*χ*2 = [ (ad - bc) (ad - bc) (a + b + c + d) ] / [ (a + b) (c + d) (b + d) ( a + c) ]
